# Deaths of Distinguished Physicians

**Published:** 1851-05

**Authors:** 


ARTICLE III.

Deaths of Distinguished Physicians.—The year 1850 has been fatal beyond all measure to medical science in France. Nearly all that remained to us illustrious in every department of the science has passed away. In chemistry Gay Lussac; in surgery, Marjorlin and Blandin; in comparative anatomy, de Blainville; in medicine, Fouquier, Royer-Collard, Capuron, Prus, and a host of others. To this long catalogue is to be added the name of M. Leuret, physician to the hospital of Bicetre. M. Leuret was one of those persons, now so rare, who pursue their career without noise or ostentation; therefore, notwithstanding the extent and variety of his knowledge, the remarkable clearness of his judgment, and his personal value, it was long before M. Leuret enjoyed that degree of public estimation to which his peculiar talents so justly entitled him. He wanted ambition, and was consequently inactive. Even his great work on the Comparative Anatomy of the Nervous system still remains unfinished.

The peculiar doctrines of M. Leuret on the moral treatment of insanity are well known. They met with obstinate opposition in France, and were the principal cause of the little public success which their professor obtained. On the other hand, M. Leuret was one of the most effective opponents of phrenology, and his profound knowledge of the comparative anatomy of the brain enabled him to overthrow many a brilliant theory, which seemed inexpugnable when applied to man alone.

Naegele, the celebrated Professor of Midwifery of Hiedelberg, and Langenbech, the no less distinguished Professor of Anatomy and Surgery at Gottingen are dead. They died on the same day; Naegele at the age of 72, Langenbech at 75. Naegele was born at Dusseldorff and studied at Strasbourg and Paris. Langenbech, at his own expense, erected and endowed at Gottingen a Surgical Hospital and Anatomical Theatre.—*N. Y. Register.*

